# Evaluation of the Interphase-Related Cycling Stability of Thin-Film Amino- and Hydroxy-Substituted Anthraquinone Electrodes for Sodium-Ion Batteries

**DOI:** 10.1021/acsaem.5c03498

**Published:** 2026-01-21

**Authors:** Victoria Greussing, Daniel Werner, Dominik Wielend, Cristian Vlad Irimia, Elisabeth Leeb, Martin Ciganek, Jozef Krajčovič, Mihai Irimia-Vladu, Engelbert Portenkirchner

**Affiliations:** † Institute of Physical Chemistry, 27255 University of Innsbruck, Innsbruck 6020, Austria; ‡ Linz Institute for Organic Solar Cells (LIOS), Institute of Physical Chemistry, 27266 Johannes Kepler University Linz, Linz 4040, Austria; § Competence Center CHASE GmbH, Hafenstrasse 47−51, Linz 4020, Austria; ∥ Brno University of Technology, Institute of Chemistry and Technology of Environmental Protection, Brno 61200, Czechia

**Keywords:** anthraquinone, SIB, anthraquinone derivative, organic electrodes, carbonyl compounds, sodium-ion batteries, thin-film electrodes

## Abstract

This study investigates sustainable approaches to designing organic cathode materials for sodium-ion batteries, aiming to replace traditional metal-based electrodes. Organic materials present a promising alternative due to their lower environmental impact, supply chain stability, and tunable electrochemical properties. In this work, the electrochemical performance of 12 commercially available amino- and hydroxy-substituted anthraquinone derivatives, including several naturally occurring compounds, was systematically evaluated in sodium-ion battery systems. By focusing on readily available commercial materials, this study identified the most stable and effective candidates for organic cathodes in sodium-ion batteries. Notably, the majority of these derivatives have never been tested in galvanostatic cycling in either lithium or other post-lithium battery systems. Through systematic testing, challenges such as high solubility and limited redox reactivity were addressed, demonstrating how careful material selection can yield high-performance, long-cycle-life organic cathodes. The performance of these materials was found to be strongly influenced by their solubility in the electrolyte as well as their structural and electronic properties, including electron-accepting capabilities and sodium coordination behavior. Among the studied materials, 1,8-dihydroxy-anthraquinone and 1,8-diamino-anthraquinone demonstrate superior cycle stability, maintaining 72% and 73% capacity retention, respectively, over 100 charge–discharge cycles, followed by 1,5-diamino-anthraquinone and 1-hydroxy-anthraquinone with 64% and 66%. These findings not only advance the development of organic cathode materials for sodium-ion batteries but also highlight the potential of sustainable material choices to enable scalable and environmentally friendly energy storage solutions, supporting the transition to a greener energy future.

## Graphical abstract

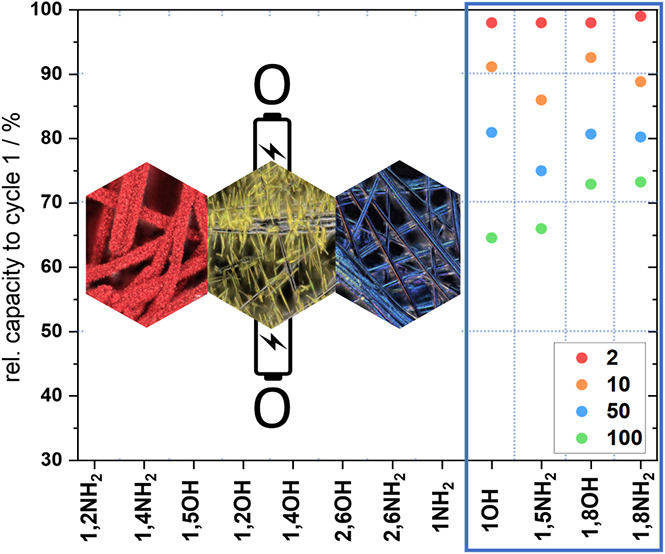

## 1 Introduction

In the 1970s and 1980s, scientific efforts began to investigate Li-ion batteries (LIB) and sodium-ion batteries (SIB) for their potential applicability as energy storage devices.^1^ Due to the rapid success of Li-ion battery technology, research into Na-ion-based batteries was largely abandoned^2^ but has been resumed again because of the natural abundance and widespread distribution of Na worldwide, in contrast to Li.^3–5^ By now, the first SIBs are already commercialized.^6^ With the research of SIBs and other post-Li-ion batteries, sustainability and recyclability are gaining importance.^7,8^ One material class having the potential for eco-efficient, low-cost, energy-efficient, and sustainable so-called “green” electrodes is organic semiconducting materials.^9–12^

One particularly interesting organic semiconducting material is anthraquinone (AQ). AQ and other quinones have a para-benzoquinone unit, which is ideal for charge storage via an enolization-type ion-coordination mechanism.^13–15^ As seen in Figure 1a, during the electrochemical reduction of the carbonyl groups, the Na-ions associate with the negatively charged oxygen atoms and dissociate upon oxidation, resulting in a reversible enolization-type redox reaction.^16,17^ Beyond their redox tunability, AQs can also be used to catalyze electrochemical reactions such as oxygen reduction reactions, showing their usefulness not just in terms of electrical charge storage but also in chemical energy storage systems. This dual functionality highlights their versatility as active materials in a range of energy conversion and storage applications.^18–20^

**Figure 1:**
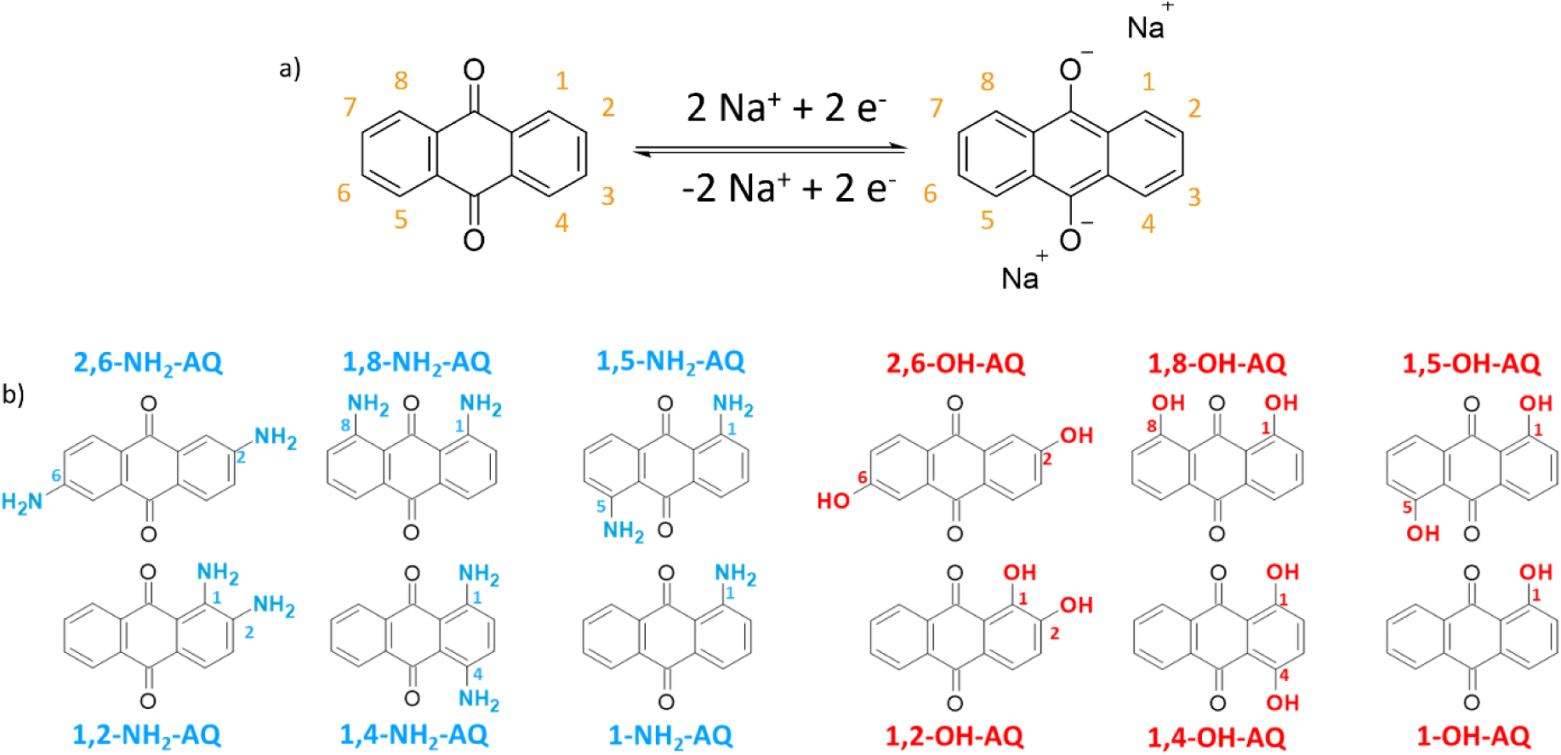
a) AQ and its possible substitution positions are highlighted in orange, along with the carbonyl redox reaction for Na-ion storage and release, and b) all the used AQ derivatives are shown on the left side with blue amino-substitution groups and on the right side with red hydroxy-substitution.

AQs, among other hydroxylated and methylated derivatives, are naturally occurring in numerous plant species, such as lichens and fungi, and have also been detected in insects.^21^ They were widely used as natural colorants, usable for dyes and pigments^22^ and AQ with amino groups can be found in inks and toners,^23^ highlighting their industrial relevance and availability. Among the AQs investigated here, the hydroxylated ones, i.e., 1,2-dihydroxy-AQ (alizarin), 1,4-dihydroxy-AQ (quinizarin), 1,5-dihydroxy-AQ (anthrarufin), 1,8-dihydroxy-AQ (chrysazin), and 2,6-dihydroxy-AQ (anthraflavic acid), have been employed extensively in the coloring and painting of garments by mankind throughout its history.^24,25^

In addition to their application as dyes and colorants, AQ and its derivatives have also exhibited promising properties as cathode materials for SIBs.^15,26,27^ However, the cycling stability is limited due to high solubility in polar organic electrolytes and chemical degradation.^16,28,29^ Furthermore, the cycling performance is also limited by aggregating into dimers, trimers, and other polyaggregates, which reduces the available storage sites for Na-ions and consequently prevents an ideal two-electron storage per molecule.^30^ On the other hand, aggregation may mitigate dissolution, which can extend cycle-life performance.^31^

When AQ is purified, it tends to form highly ordered crystalline structures with strong intermolecular π–π interactions.^32^ With the addition of substitution groups to the AQ, the electrode characteristics can be tuned, and this may also positively influence the electrochemical stability due to differences in inter- and intramolecular hydrogen bonds in the system.^33^

Interestingly, when purified AQ derivatives^34^ were evaporated onto carbon paper substrates, a wide range of distinct morphologies occurred—from needle-like and crystalline deposits within the fibers to smooth continuous films—depending on the respective anthraquinone derivative. These differences in observed morphologies and interfaces are expected to play a key role in the electrochemical performance of the materials. Interestingly, the pronounced differences in morphology and interfacial characteristics did not translate into noticeable variations in electrochemical performance, contrary to common expectations regarding their influence. However, these differences in morphology and interfacial characteristics did not lead to a significant variation in electrochemical performance.

In recent studies, several AQ derivatives with amino- and hydroxy-substitution were investigated, combining experimental and computational methods. One study focused on the calculation of electrochemical reduction potentials of several AQ derivatives via an innovative DFTB approach and correlated against experimental data.^35^ In a recent work, our group investigated the redox potential of hydroxy- and amino-AQs, demonstrating a significant anodic and cathodic shift, respectively.^36^ Moving toward carbon dioxide (CO_2_) mitigation, one collaborative study focused on the influence of the different substituents of AQ on possible electrochemical CO_2_ capture performance and spectroelectrochemical data. The latest collaborative work applied and combined the knowledge gained in the aforementioned studies and reports the efficient use of an immobilized AQ polymer for electrochemical CO_2_ capture and release.^37^

Based on these publications, in this study, six naturally occurring hydroxy-AQ derivatives and six amino-AQ derivatives were investigated as potential cathode materials in SIBs, as shown in Figure 1b. Furthermore, these samples were analyzed using an optical microscope, scanning electron microscopy (SEM), thermogravimetric analysis (TGA), attenuated total reflection Fourier transformed infrared spectroscopy (ATR-FTIR), cyclic voltammetry (CV), and galvanostatic charge/discharge cycling with potential limitations (GCPL).

## 2 Methods

### 2.1 Electrode Preparation

Preparation of 1,8-diamino-anthraquinone (1,8-NH_2_-AQ): This compound was prepared according to a literature procedure.^38^ A solution of sodium sulfide hydrate (3.148 g, 25.70 mmol) in distilled water (68 mL) was added to a solution of 1,8-dinitroanthracene-9,10-dione (1,8-NO_2_-AQ, 1.008 g, 3.38 mmol) in ethanol (20 mL) at 25 °C. After that, the reaction mixture was heated to reflux (∼115 °C in an oil bath) and stirred for 18 h. Then, the reaction mixture was cooled to 20 °C and poured into ice/water mixture (100 mL). The resulting precipitate was filtered, washed with distilled water (2 × 40 mL), and dried in a vacuum desiccator at 50 °C for 24 h. Finally, the crude material was recrystallized from ethanol (15 mL) to obtain 1,8-NH_2_-AQ as a dark red needlelike solid material (0.768 g, 96.1%). Analytical data are in agreement with those previously reported in the literature. The data can be found in the Supporting Information.

All the AQ derivatives—other than 1,8-NH_2_-AQ—employed in this study were scrupulously purified by two consecutive vacuum sublimation steps.^34^ The purified compounds were then placed in a thermal evaporator, and thin films of AQ and its substituted analogues were deposited via sublimation under reduced pressure (10^–6^ mbar). Thin-film electrodes were prepared by thermally evaporating purified hydroxy- and amino-substituted AQ derivatives onto carbon-carrier substrates (carbon paper, CP). Circular carbon paper discs (17 mm in diameter) were punched out from MGL370 carbon sheets with a 0.3 mm thickness to function both as the substrate and current collector.

### 2.2 Electrode Morphology and Spectroscopy

The coated fibers and the overall morphology of the electrodes were observed with a KEYENCE VHX-7000 digital microscope.

The morphology of the prepared electrodes was also analyzed using a SEM. A JEOL JSM-6360LV scanning electron microscope was operated under high vacuum settings and an acceleration voltage of 7.0 kV.

The mass of the sublimated AQ derivatives was determined using TGA measurements performed with a NETZSCH STA 449F3 Jupiter TGA/DSC setup. The weight loss of the electrodes was measured in an argon atmosphere over a temperature ramp from 25 to 700 °C at a rate of 10 °C/min.

The electrodes were additionally analyzed with ATR-FTIR. For this purpose, the coated side was pressed against the ZnSe crystal under ambient conditions. For the measurements, the apparatus Bruker Vertex 80 with an ATR accessory was used, and then a range of 4000–400 cm^–1^ with 32 scans and a resolution of 2 cm^–1^ was measured.

### 2.3 Battery Assembly and Electrochemical Measurements

Electrochemical testing was carried out in a three-electrode ECC-ref Cell (El-Cell) using a Biologic VMP3 Potentiostat at room temperature. The working electrodes had a diameter of 17 mm. Na (VWR, 99.5%), stored in paraffin oil, functioned as both the counter electrode (Ø = 17 mm) and reference electrode, while a glass fiber separator disc (Ø = 18 mm, thickness 1.55 mm, El-Cell) was used as the separator. The electrolyte (500 μL), sourced from Solvonic (99% purity), consisted of 1 M sodium bis­(fluorosulfonyl)­imide (NaFSI) dissolved in a 1:1 (v/v) mixture of ethylene carbonate (EC) and dimethyl carbonate (DMC). Cell assembly was conducted in an argon-filled glovebox (UNI-lab, MBraun) with water and oxygen levels kept below 0.1 ppm.

## 3 Results

### 3.1 Analysis and Morphology

Twelve different AQ derivatives (Table 1) were tested in SIB half-cells. The electrodes are named x,y–OH-AQ and x,y–NH_2_-AQ, depending on the substitution group, with x and y being the substitution positions.

**Table 1:** List of the 12 Tested Substances with Their Abbreviations Used in This Publication^a^

Hydroxy AQ:		Amino AQ:	
Abbreviation		Abbreviation	
1-OH-AQ	1-Hydroxy-anthraquinone	1- NH_2_-AQ	1-Amino-anthraquinone
1,2-OH-AQ	1,2-Dihydroxy-anthraquinone	1,2- NH_2_-AQ	1,2-Diamino-anthraquinone
1,4-OH-AQ	1,4-Dihydroxy-anthraquinone	1,4- NH_2_-AQ	1,4-Diamino-anthraquinone
1,5-OH-AQ	1,5-Dihydroxy-anthraquinone	1,5- NH_2_-AQ	1,5-Diamino-anthraquinone
1,8-OH-AQ	1,8-Dihydroxy-anthraquinone	1,8- NH_2_-AQ	1,8-Diamino-anthraquinone
2,6-OH-AQ	2,6-Dihydroxy-anthraquinone	2,6- NH_2_-AQ	2,6-Diamino-anthraquinone

a The mono- and disubstituted hydroxy-anthraquinones are listed on the left, and the mono- and disubstituted amino-anthraquinones are listed on the right side.

The chemical composition and structures of the different AQ compounds were analyzed with FTIR (Figures S1–S2), optical microscopy (Figure 2 and Figure S3), and SEM (Figures S4–S5). Both optical imaging and SEM were employed to complement each other. SEM images offer significantly higher resolution and depth of field compared with optical microscopy, enabling detailed visualization of surface morphology at the nanoscale; however, they lack intrinsic color information, which can be advantageous in optical techniques, especially for color-rich pigment molecules like AQs. Figure 2 shows optical microscopy images (top view) of six selected AQ-derivative thin-film electrodes.

**Figure 2:**
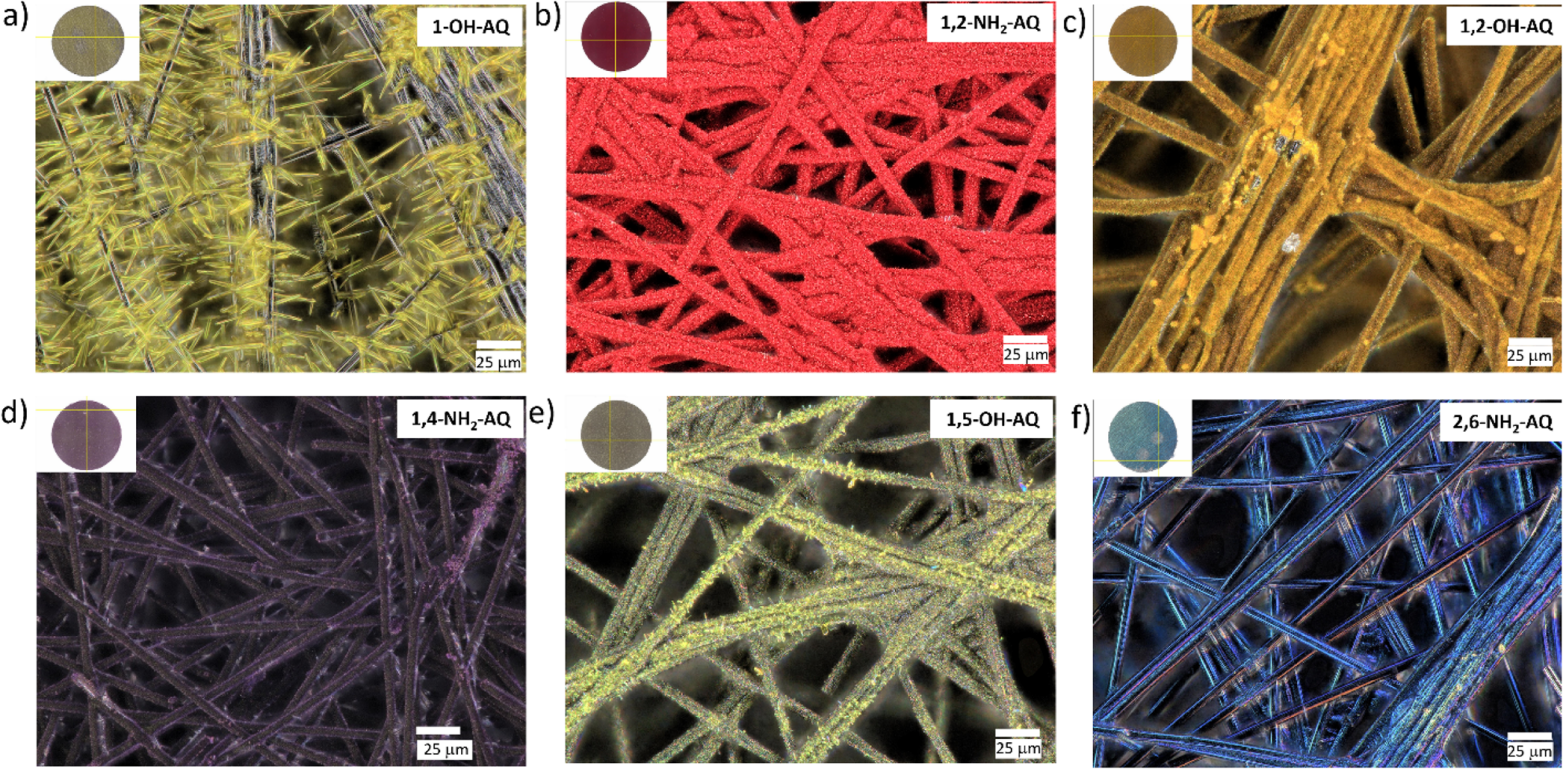
Optical microscopic images of six different AQ derivatives on a CP substrate: a) 1-OH-AQ, b) 1,2-NH_2_-AQ, c) 1,2-OH-AQ, d) 1,4-NH_2_-AQ, e) 1,5-OH-AQ, and f) 2,6-NH_2_-AQ. In all images, an overview of the total electrode is displayed in the upper left corner, with a yellow cross marking the exact location where the image was captured. The magnification for all images was set to 1000×, and a scale bar is provided in the bottom right corner.

These six active materials were chosen to highlight the different interphases and morphologies in each of the fabricated AQ-CP composite electrodes. 1-OH-AQ (Figure 2a) forms several μm long yellow needles growing at a right angle from the subjacent carbon fibers, which are distinctly visible in gray. Differently, 1,2-NH_2_-AQ, 1,2-OH-AQ, and 1,4-NH_2_-AQ (Figure 2b-d) are characterized by small crystals covering the fibers completely. 1,5-OH-AQ (Figure 2e) displays a continuous yellow-green film with several small crystals on top, indicating different heights at different spots. The image for 2,6-NH_2_-AQ (Figure 2f) exhibits a blue, closed film with no sign of any sort of needles or crystals on top. These findings emphasize the different crystallization characteristics of AQ derivatives on the carbon substrates significantly more compared to previous reports.^26,36^

In addition, the findings from optical microscopy were further confirmed and complemented by SEM imaging. With the SEM images (Figures S4–S5), the presence of these different morphologies can be observed. It is particularly noteworthy that the needles in 1,2-NH_2_-AQ, 1,4-NH_2_-AQ, 1,8-NH_2_-AQ, and 1-OH-AQ (Figure S4b, c, e and Figure S5a) are clearly discernible. Among the electrodes that exhibit an almost perfectly uniform thin film are 2,6-NH_2_-AQ and 2,6-OH-AQ (Figure S4f, S5f). No distinguishable features can be observed in the SEM images. However, the presence of the thin film was substantiated through optical microscopy and IR analysis.

Building on these pronounced differences in crystal shape and surface morphology, one would expect distinct interphase properties at the electrode–electrolyte interface, likely resulting in significant variations in electrochemical performance. Surprisingly, however, follow-up electrochemical experiments revealed only minor differences between the active materials, indicating that the observed morphological variations did not translate into the anticipated performance disparities.

### 3.2 Electrochemical Characterization and Battery Testing

A previous study of our group focused on the tunability of the redox potential upon side group substitution of AQ with either OH or NH_2_ groups. It has been demonstrated that by adding either two amino or two hydroxy groups to AQ the reduction potential shift between the molecules can be up to 700 mV in solution and 440 mV in a half-cell battery setup.^35,36^ The CVs for the AQ derivatives measured in solution were as expected, with two reduction peaks and two oxidation peaks for the AQ derivatives. These two peak pairs are characteristic for the two carbonyl groups of AQ and its derivatives. When it comes to battery measurements using AQ-CP composite electrodes, the CVs tend to become much more complex and consequently more challenging to interpret, as presented in the following Figure 3.

**Figure 3:**
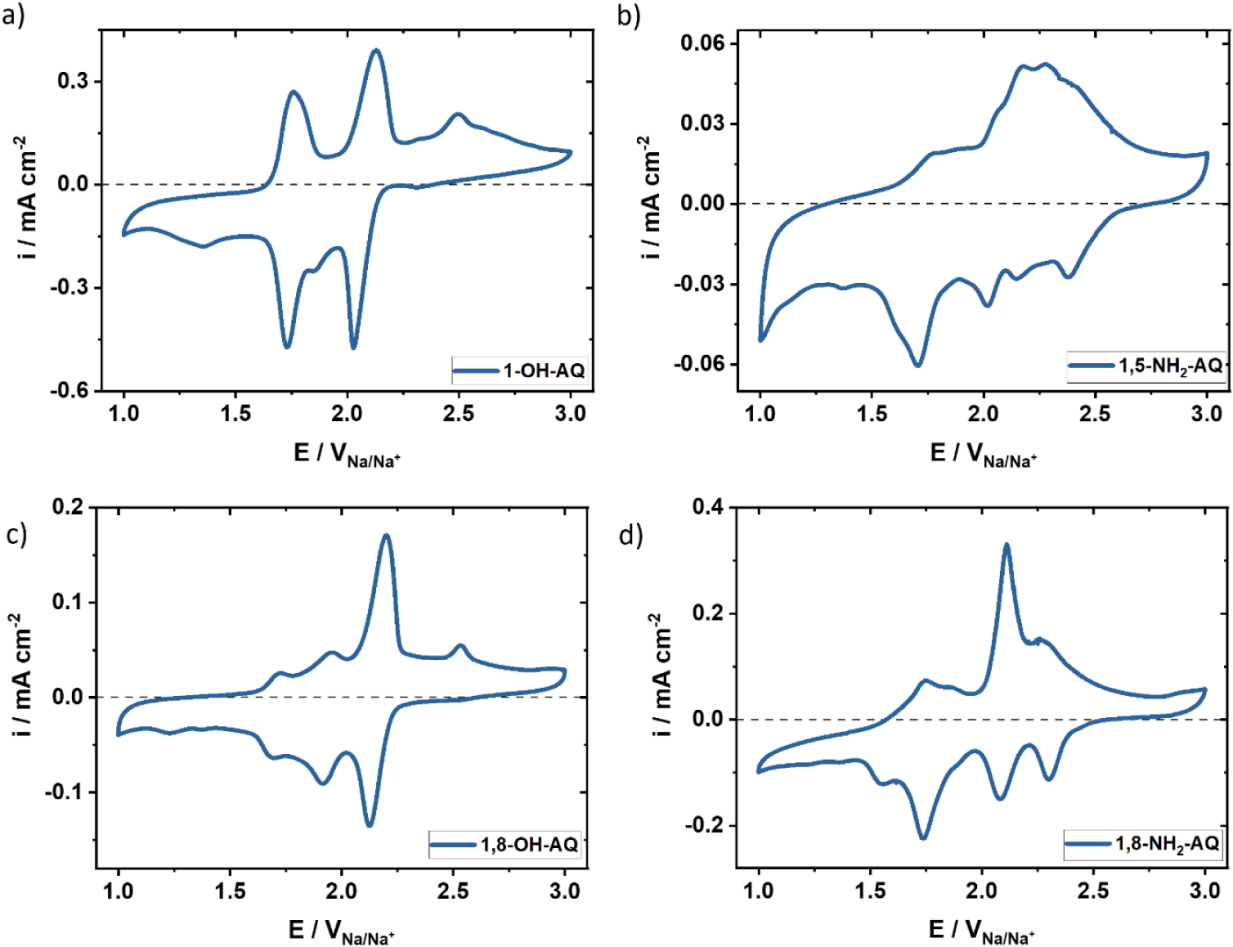
CV measurements of a) 1-OH-AQ, b) 1,5-NH_2_-AQ, c) 1,8-OH-AQ, and d) 1,8-NH_2_-AQ thin-film electrodes with a scan rate of 5 mV s^–1^ in NaFSI dissolved in a 1:1 (v/v) mixture of EC and DMC.

Figure 3 depicts CV measurements of the four most promising derivatives, namely, 1-OH-AQ, 1,5-NH_2_-AQ, 1,8-OH-AQ, and 1,8-NH_2_-AQ. The thin-film electrodes were measured in the potential range between 1.0 and 3.0 V vs Na/Na^+^, at a scan rate of 5 mV s^–1^, showing the 3rd cycle. For comparison, cycle 1 for each of these four derivatives can be found in Figure S6. As reported earlier,^12,39^ the CP is electrochemically inactive in this potential window.

The 1-OH-AQ CV (Figure 3a) exhibits two main peak pairs at 2.06/2.13 V and 1.73/1.79 V. Additionally, small peaks are observed at 1.85 and 1.35 V in the reduction and at 2.49 V for the oxidation scan. For 1,5-NH_2_-AQ (Figure 3b), the CV measurement is more complicated due to at least five distinctive reduction peaks and one small and one very broad oxidation peak. Most of these peaks appear after the first reduction (first cycle in Figure S6a), which indicates dimer formation and or significant structural change during the cycling.^15^ In Figure 3c, three main peak pairs of 1,8-OH-AQ are identified at potentials of 1.56/1.72 V, 1.84/1.96 V, and 2.03/2.31 V. In addition, a small reduction peak at 1.32 V and an oxidation peak at 2.54 V are observed from the first cycle. These peaks are likely related to dimerization, which occurs during the evaporation or condensation processes during electrode preparation. In Figure 3d the CV of 1,8-NH_2_-AQ is also characterized by three main peak pairs at 1.74/1.74 V, 2.09/2.10 V, 2.30/2.34 V, and one small reduction peak at 1.53 V. Especially for 1,8-NH_2_-AQ the difference between the first cycle and the subsequent cycles is quite significant. In cycle 1, there are two reduction peaks at 1.51 and 1.73 V with similar current maxima and three oxidation peaks at 1.75, 2.11, and 2.33 V. These peaks are easily distinguished and do not overlap at all, while in cycle three (Figure 3d) it becomes challenging to clearly identify the exact number of peaks. This strongly suggests a reorganization and/or dimerization of the AQ derivative.^26^ Another possible explanation for the presence of multiple peaks could be the polymerization of the amino derivatives during the redox reactions.^40,41^ In particular, amino-AQ derivatives are known to polymerize in strongly acidic aqueous solutions.^37,42^ Therefore, it is conceivable that polymerization may also occur in aprotic electrolytes with high Na^+^ concentrations, potentially resulting in additional peaks during the cycling of the battery electrodes.

After the CVs were recorded, GCPL was performed to evaluate the electrochemical Na-ion storage capacity performance. The mass of the active AQ material was evaluated beforehand with TGA (Figures S7–S9). A total of 100 GCPL cycles were conducted to assess the stability and capacity retention of the amino- and hydroxy-substituted AQs. The theoretical capacity for x,y-NH_2_-AQ is 225 mA h g^–1^, while for x,y–OH-AQ it is 223 mA h g^–1^, based on the molecular mass. The galvanostatic charge/discharge performance of these four AQ derivative thin-film electrodes, investigated with a 1C rate, is shown in Figure 4, while measurements for the other derivatives can be found in the Supporting Information (Figures S10–S11).

**Figure 4:**
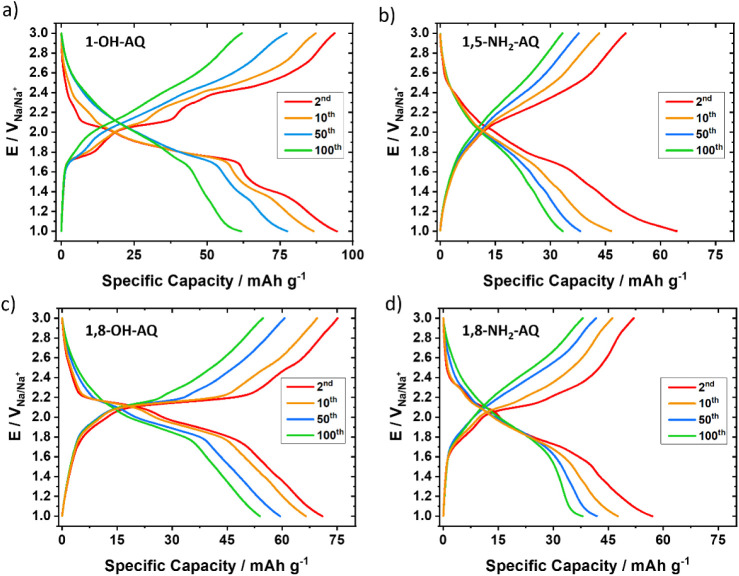
Galvanostatic charge/discharge performances of a) 1-OH-AQ, b) 1,5-NH_2_-AQ, c) 1,8-OH-AQ, and d) 1,8-NH_2_-AQ thin-film electrodes for 100 cycles with an applied constant current of 1C. Images show the 2nd, 10th, 50th, and 100th cycles, respectively.

The 1-OH-AQ (Figure 4a) thin-film electrode showed a discharge capacity of 95 mA h g^–1^ in the second cycle, while decreasing to only 62 mA h g^–1^ after 100 cycles. The charge/discharge behavior reveals three distinct plateaus at 2.1 V, at 1.8 V and at 1.4 V. For the 1,5-NH_2_-AQ (Figure 4b) thin-film electrode, the discharge capacity at the second cycle was 64 mA h g^–1^ and after 100 cycles 33 mA h g^–1^. The thin-film electrode with 1,8-OH-AQ (Figure 4c) started with 71 mA h g^–1^ and decreased to 54 mA h g^–1^ from cycle 2 to cycle 100. For 1,8-NH_2_-AQ (Figure 4d) the discharge capacity amounts to 57 mA h g^–1^ after the second cycle and after 100 cycles to 38 mA h g^–1^.

To further analyze the long-term cycling performance, the capacity retention was plotted along with the calculated Coulomb efficiency against the cycle number in Figure 5 (and Figures S12–S13). For all derivatives, the capacity gradually decreases upon cycling. The specific capacity reduces more rapidly during the first ten cycles, after which the capacity retention stabilizes.

**Figure 5:**
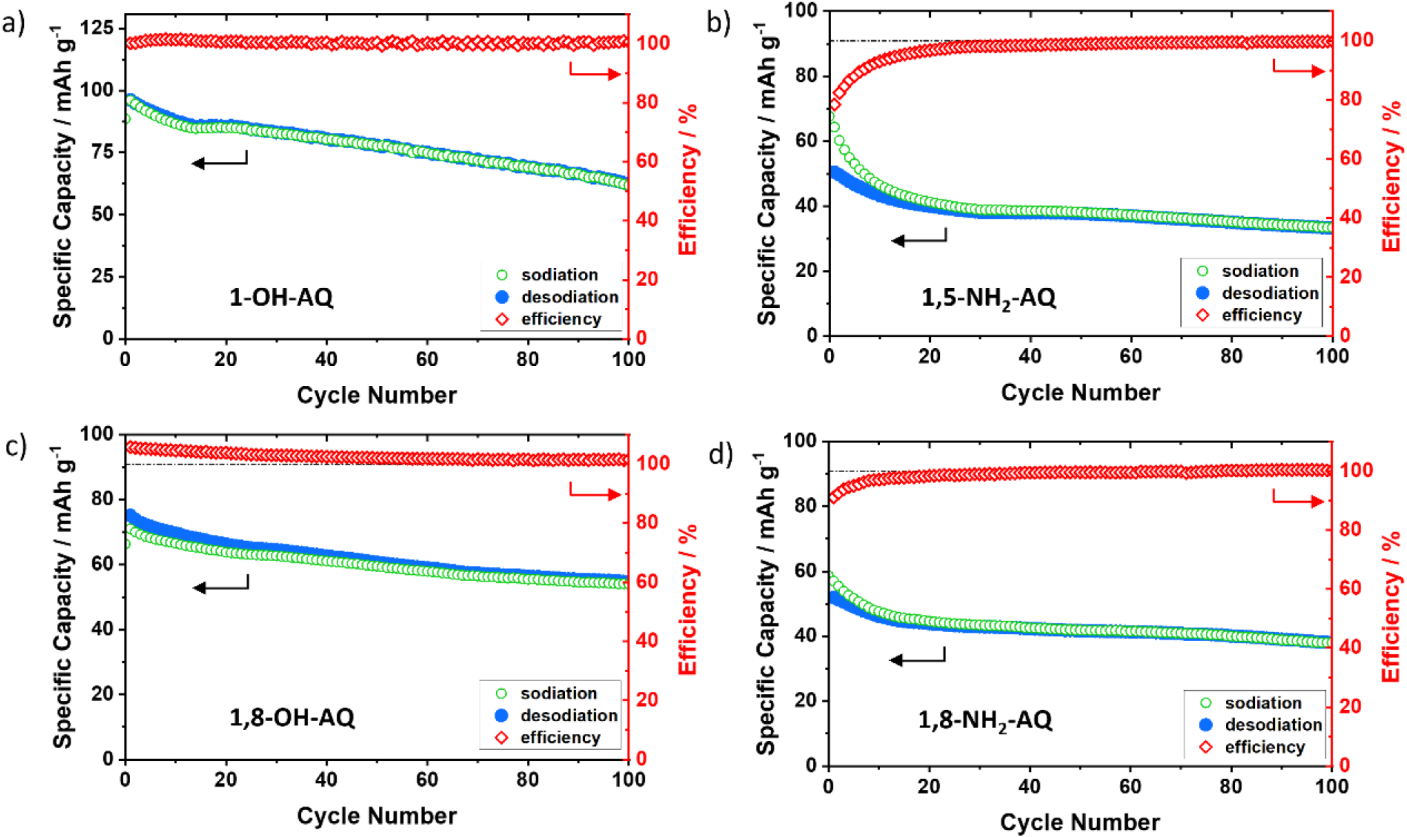
Capacity retention of a) 1-OH-AQ, b) 1,5-NH_2_-AQ, c) 1,8-OH-AQ, and d) 1,8-NH_2_-AQ thin-film electrodes for 100 cycles with an applied constant current of 1 C.

Interestingly, for 1,8-OH-AQ (Figure 5c) the Coulomb efficiency is above 100% efficiency, since charge transfer for the desodiation capacities is higher than that for the sodiation. This unusually high efficiency likely arises from a structural reorganization of the film rather than additional Na-ion storage,^26^ as supported by the continuously decreasing capacity over subsequent cycles. The underlying reason why this phenomenon occurs specifically for 1,8-OH-AQ, as well as for some cycles of 1-OH-AQ, remains unclear and requires further investigation.

Overall, the 12 derivatives show very interesting yet distinct results in the GCPLs and accordingly in the capacity retention plots. For a better comparison of their cycling stability, the Na-ion storage capacities were normalized to the first charge/discharge cycle of each electrode. The relative capacity to cycle 1 was then plotted for cycles 2, 10, 50, and 100, as shown in Figure 6. A summary of the capacities at cycles 1 and 100, as well as the calculated capacity retention, is provided in the Supporting Information (Table S1).

**Figure 6:**
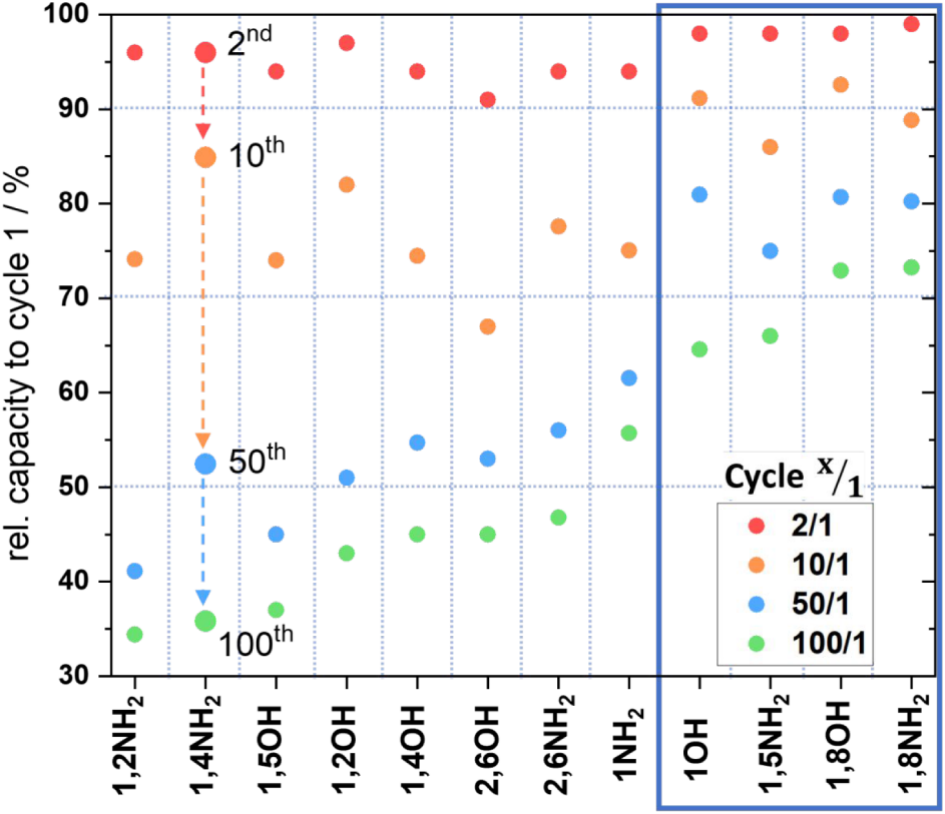
Relative capacity of cycle 2 (red), cycle 10 (orange), cycle 50 (blue), and cycle 100 (green) in comparison to cycle 1 for 12 AQ derivatives. The four derivatives with the highest relative values after 100 cycles—shown in the blue frame—are 1-OH-AQ, 1,5-NH_2_-AQ, 1,8-OH-AQ, and 1,8-NH_2_-AQ.

Figure 6 summarizes the galvanostatic cycling characteristics of the different derivatives and how the capacity decreases for all of the tested active materials upon extended cycling. From left to right, the AQ derivatives are depicted with the lowest values to the highest values after 100 cycles. 1,2-NH_2_-AQ, 1,4-NH_2_-AQ and 1,5-OH-AQ retain only approximately 35% of their initial capacity; therefore, these derivatives suffer the highest capacity loss among all the measured active materials. However, these long-term cycling performances are still in the same range as the parent molecule AQ, with 34% capacity retention after 100 cycles for an AQ thin-film electrode, as reported in our previous work.^26^ Notably, all other tested derivatives retain a relative capacity after 100 cycles that is higher than that of AQ itself.

Furthermore, for 1,2-OH-AQ, 1,4-OH-AQ, 2,6-OH-AQ, and 2,6-NH_2_-AQ only 43%, 45%, 46%, and 47%, respectively, of the initial capacity remains after 100 cycles. 1-NH_2_-AQ remains, with 55%, a slightly higher capacity after cycling. Four derivatives demonstrate a clearly superior capacity retention in this series of measurements, highlighted with the blue frame. These are 1-OH-AQ, 1,5-NH_2_-AQ, 1,8-OH-AQ, and 1,8-NH_2_-AQ. The latter shows the highest relative capacity after cycle 2 and continues this trend for cycles 10 and 50, with more than 60% of the initial capacities left after 100 cycles. With a remaining 73% of its capacity compared to cycle 1 after 100 cycles, 1,8-NH_2_-AQ is the best-performing in our series of molecules. The focus in the following discussion will be on the four best-performing derivatives with the highest relative capacity remaining after cycling.

The poor cycling stability of AQ and other organic compounds is well documented; its high solubility in traditional carbonate-based electrolytes leads to a fast decay of capacity.^43,44^ This solubility issue is also observed in the separators following the disassembly of the batteries, which are colored yellow, orange, red, or purple according to the AQ derivative used (Figure S14). Due to the (high) solubility in the electrolyte, a loss of active material from the electrode occurs. Several studies have worked on improving the stability by adding functional groups with varying degrees of success.^45,46^ The present study demonstrates that the position of the added functional group is also of significance. It seems that the 1-, 1,5-, and especially the 1,8-position next to the carbonyl groups are better for cycling stability, but interestingly, the 1,4-position does not seem to have this effect. However, as the pristine AQ molecule has the lowest solubility in common organic solvents compared to all of these derivatives, this study clearly demonstrates the benefit of hydroxy and amino substitution, as all 12 derivatives show better capacity retention compared to bare AQ.^47,48^

The morphology of the electrodes in Figure 2 also shows interesting conclusions when compared with these results. The 1-OH-AQ (Figure 2a) has one of the highest capacity retention values among the investigated electrode materials after cycling, which is remarkable, considering that the 1-OH-AQ did not form a continuous thin film but rather numerous relatively long needles up to 50 μm compared to the small crystals or flat film in the sub μm range. The derivatives 1,2-NH_2_-AQ, 1,2-OH-AQ, 1,4-NH_2_-AQ, and 1,5-NH_2_-AQ (Figure 2b-e) all show similar morphology and have less than 50% of their capacity left after 100 cycles. But astonishingly, 2,6-NH_2_-AQ, which shows an almost perfect thin film covering the subjacent carbon fibers, would be expected to have a good cycling stability accordingly; however, it does not. Although the evaporated AQ thin films possess a high degree of structural versatility, this does apparently not correlate to their cycling capability and stability, which depends on morphologies and interphases.

Based on these observations, it can be assumed that AQ derivatives undergo structural changes upon cycling, which was already suggested in previous works for AQ^26^ and PTCDI,^39^ with the nature and extent of this reorganization potentially depending on their initial morphology. However, the results reveal no clear correlation among the original electrode morphology, the extent of structural reorganization, and the resulting electrochemical cycling performance. While some derivatives with discontinuous or needle-like morphologies retain a relatively high capacity and almost no changes in CV regarding the shape, potential, or number of the redox peaks from the first to the subsequent cycles, as for 1-OH-AQ, others with seemingly ideal thin-film coverage show poor cycling stability. This indicates that additional molecular-level interactions, such as differences in π–π interaction energies, (de)­sodiation mechanisms, or the ability to form hydrogen bonds, may strongly influence both the restructuring behavior and the stability of the active material during cycling. These interactions, which are determined by the type and position of the functional groups, likely play a more dominant role than the initial morphology alone in dictating the long-term electrochemical performance.

## 4 Conclusion

In conclusion, amino- and hydroxy-substituted anthraquinone derivatives combine sustainability, availability, and tunable performance. Many derivatives are low-cost, commercially accessible, and, in some cases, already used industrially or naturally occurring, supporting future recyclability. AQ and its derivatives enable two single-electron transfer steps with accordingly high theoretical capacities of more than 220 mAh g^–1^, and their redox potentials can be systematically tuned via functional groups (e.g., −OH, −NH_2_), enabling optimization of operating voltage and energy density.

This study experimentally evaluated 12 commercially available AQ derivatives, which are partly derived from nature, as organic cathode materials. Among the initial screening, the most stable and effective candidates for SIBs were identified. Two derivatives with substituents located adjacent to the same carbonyl group, 1,8-OH-AQ and 1,8-NH_2_-AQ demonstrated outstanding performance, retaining more than 70% of their initial capacity after 100 with specific capacities of 71 and 54 mA h g^–1^, respectively, in the second cycle. On the other side, 1,5-OH-AQ, 1,4-NH_2_-AQ, and 1,2-NH_2_-AQ have the highest losses in capacity over 100 cycles and are the only three derivatives with a similar loss of capacity as AQ itself, which we reported as 34% after 100 cycles.^26^ The focus on this present study lies on the capacity loss in percentage and provides a detailed comparison between the mono- and disubstituted hydroxy- and amino-AQs. Furthermore, the electrodes were analyzed thoroughly with IR, SEM, optical microscopy, and by performing CVs and GCPLs, additionally analyzed with capacity retention plots.

This work addressed the ongoing challenges associated with solubility and redox reactivity, particularly for quinone-based cathodes, where capacity loss during cycling remains a critical issue. The comparative analysis between mono- and disubstituted hydroxy- and amino AQ derivatives revealed insights into the relationship between substitution patterns and electrochemical performance. However, based on our findings, it can be concluded that the microscopic film morphology does not provide a clear indication as to which electrode is more stable.

Beyond the specific findings, this study underscores the potential of commercially available AQ derivatives as sustainable and scalable cathode materials for SIBs, extending beyond their established uses as electrocatalysts in solar fuel production.^49^ However, to fully realize their potential, challenges such as solubility and long-term stability must be further addressed. Future research should focus on exploring alternative functional groups, optimizing electrolyte formulations, and testing these materials in full-cell configurations to bridge the gap between laboratory studies and practical applications.

Overall, this work contributes to the growing body of research on organic electrode materials, demonstrating that commercially available AQ derivatives hold substantial promise as environmentally friendly and high-performance energy storage solutions for batteries and beyond.

## Supplementary Material



## Notes

Synthetic procedure for 1,8-NH2-AQ; IR spectra; optical microscopy images; SEM images; first-cycle cyclic voltammograms of 1-OH-AQ, 1,5-NH_2_-AQ, 1,8-OH-AQ, and 1,8-NH2-AQ; TGA data; table of mass loading and capacities (cycle 1 and cycle 100); GCPLs and capacity retention plots (PDF)
