# Supplementary material for: Evaluation of the Interphase-Related Cycling Stability of Thin-Film Amino- and Hydroxy-Substituted Anthraquinone Electrodes for Sodium-Ion Batteries
Source: ACS Appl Energy Mater. 2026 Jan 21;9(3):1638–46. doi: 10.1021/acsaem.5c03498 (PMC12892241; doi:10.1021/acsaem.5c03498)
Supplement: Supplementary file 1 [file ae5c03498_si_001.pdf]

Supporting Information

"Evaluation of the interphase related Cycling Stability of thin-film Amino- and Hydroxy-Substituted Anthraquinone Electrodes for Sodium-Ion Batteries"

Victoria Greussing[a+*], Daniel Werner[a+], Dominik Wielend[b,c], Cristian Vlad Irimia[b], Elisabeth Leeb[b], Martin Ciganek[d], Jozef Krajčovič[d], Mihai Irimia-Vladu[b], Engelbert Portenkirchner[a*]

[a] *Institute of Physical Chemistry, University of Innsbruck, 6020 Innsbruck, Austria*

[b] *Linz Institute for Organic Solar Cells (LIOS), Institute of Physical Chemistry, Johannes Kepler University Linz, 4040 Linz, Austria*

[c] *Competence Center CHASE GmbH, Hafenstrasse 47–51, 4020 Linz, Austria*

[d] *Brno University of Technology, Institute of Chemistry and Technology of Environmental Protection, 61200 Brno, Czechia*

[+] These authors contributed equally to this work

[*] e-mail: victoria.greussing@uibk.ac.at; engelbert.portenkirchner@uibk.ac.at

Additional experimental information

Synthetic procedures

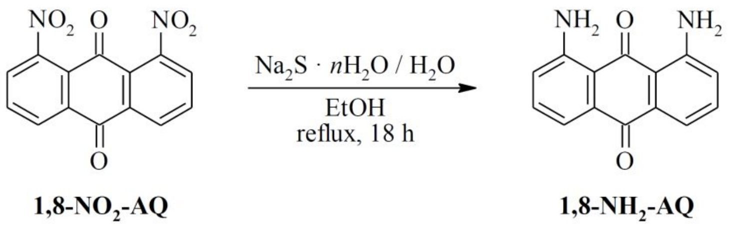

General information. 1,8-Dinitro-anthraquinone (1,8-NO_2-AQ), sodium sulfide hydrate pure ($Na_2S \cdot nH_2O$, 63.7w%), ethanol (p.a., 96%) were purchased from *Sigma-Aldrich*. All chemicals were used without further purification. ^{1}H-NMR and ^{13}C-NMR spectra were recorded from solutions in deuterated $CDCl_3$ and DMSO-d_6 solvents on FT-NMR spectrometer Bruker AVANCE™ III 500 MHz, and chemical shifts (δ) are given in parts per million (ppm) relative to tetramethylsilane (TMS) as an internal reference. Melting points were determined on a Kofler apparatus (type KB T300 equipped with an optical system with a 40× magnification) and the temperature was not calibrated. Elemental analysis of pure product was performed on a Elementar Vario Micro Cube CHNS-analyzer.

Melting point: 266 ˚C (lit.[1] melting point 265-266 ˚C). ^{1}H-NMR (500 MHz, DMSO-d_6, ppm): δ = 7.85 (s, 4H), 7.44 (dd, J = 8.2, 7.3 Hz, 2H), 7.33 (d, J = 7.3 Hz, 2H), 7.15 (d, J = 8.2 Hz, 2H). ^{13}C-NMR (126 MHz, $CDCl_3$, ppm): δ = 188.9, 184.2, 151.2, 134.5, 134.2, 123.6, 116.2, 114.5. Anal. calcd. for $C_{14}H_{10}N_2O_2$: C 70.58%, H 4.23%, N 11.76%; found: C 70.65%, H 4.11%, N 11.79%.

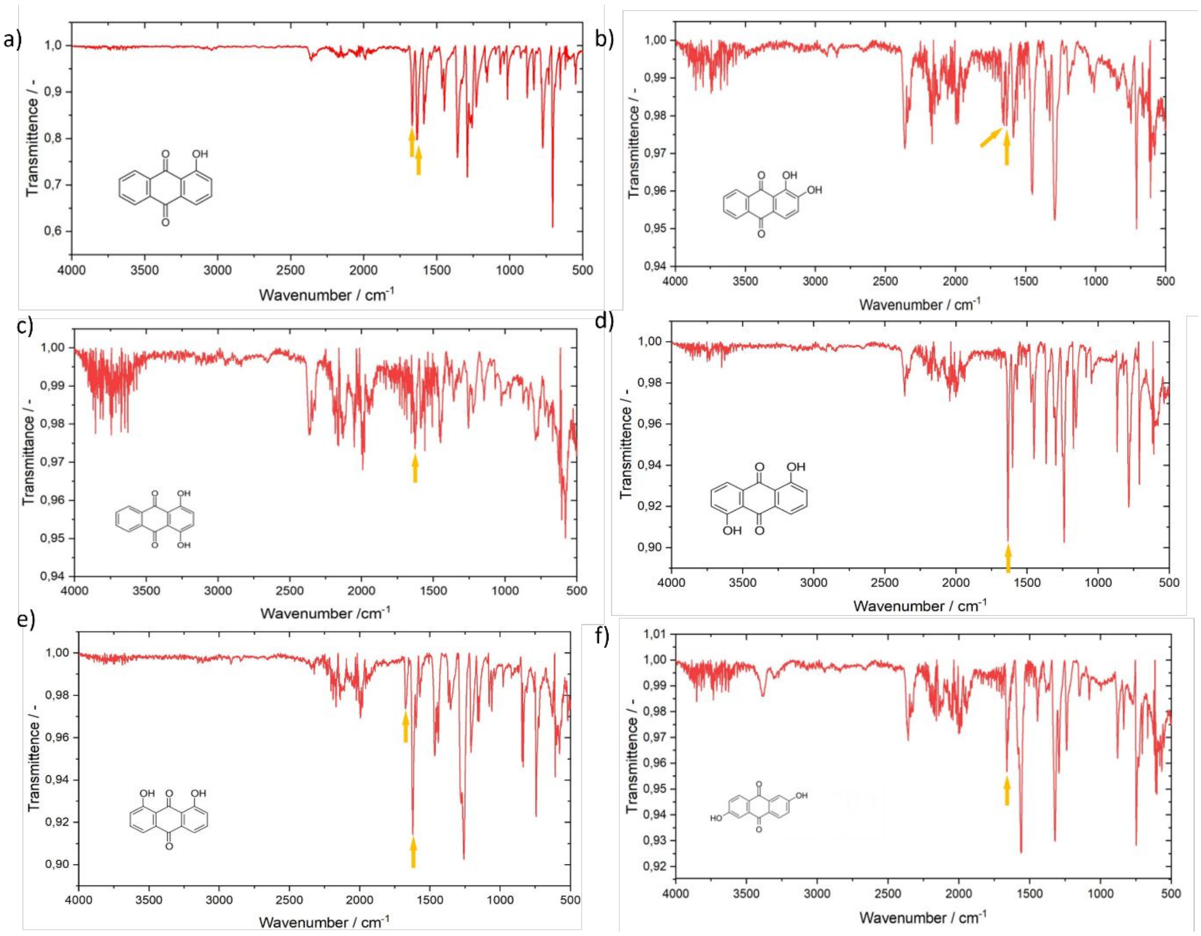

Figure S1. ATR-FTIR spectra in the range of 4000 to 500 cm^{-1} of a) 1-OH-AQ, b) 1,2-OH-AQ c) 1,4-OH-AQ, d) 1,5-OH-AQ e)1,8-OH-AQ and f) 2,6-OH-AQ with the carbonyl peaks highlighted with one or two yellow arrows, depending if there is one or two carbonyl peaks.

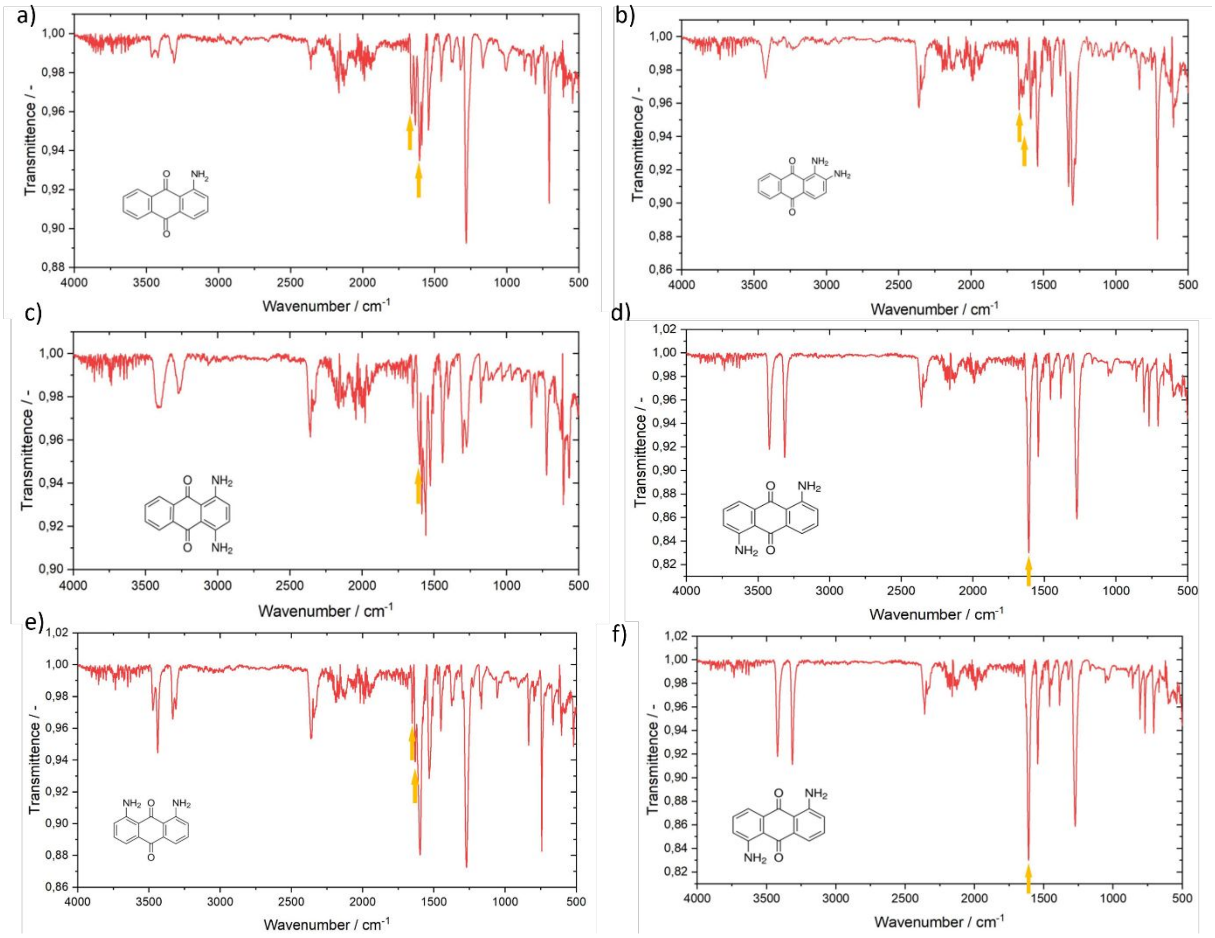

Figure S2. ATR-FTIR spectra in the range of 4000 to 500 cm^{-1} of a) 1-NH_2-AQ, b) 1,2-NH_2-AQ c) 1,4-NH_2-AQ, d) 1,5-NH_2-AQ, e) 1,8-NH_2-AQ and f) 2,6-NH_2-AQ with the carbonyl peaks highlighted with one or two yellow arrows, depending if there is one or two carbonyl peaks.

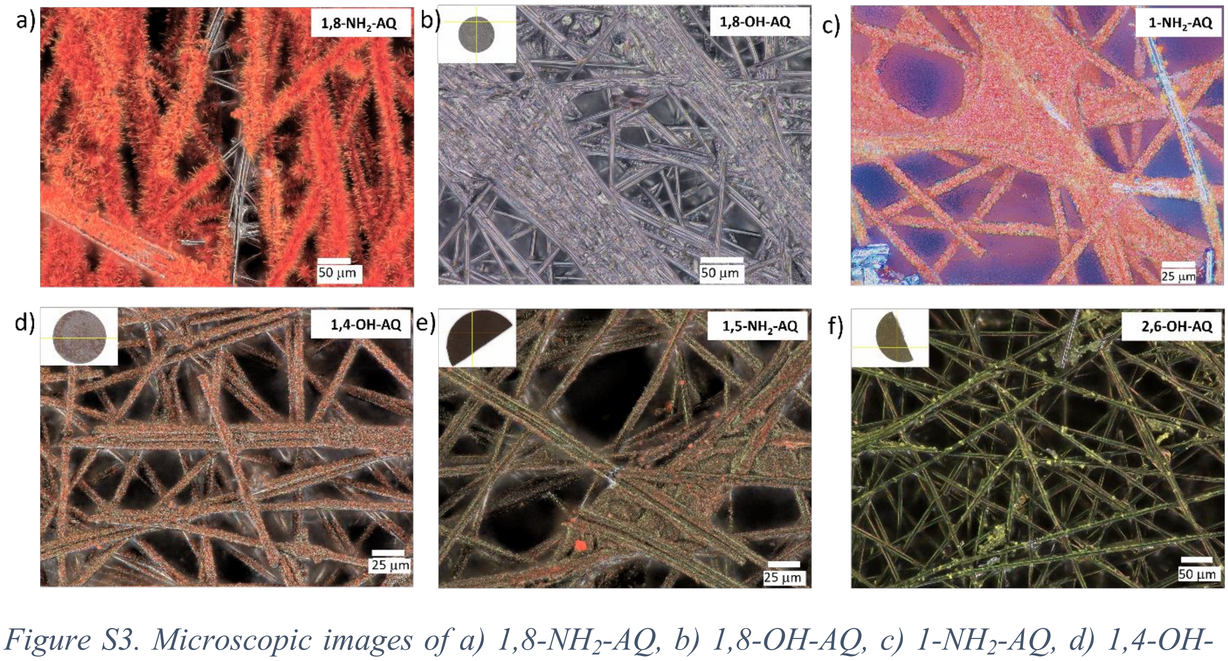

Figure S3. Microscopic images of a) 1,8-NH_2-AQ, b) 1,8-OH-AQ, c) 1-NH_2-AQ, d) 1,4-OH-AQ, e) 1,5-NH_2-AQ and f) 2,6-OH-AQ on a CP substrate. In S3b,d-f), the overview of the total electrode is displayed in the upper left corner, with two yellow lines—one horizontal and one

vertical—intersecting to mark the exact location where the image was captured. A scale bar is provided in the bottom right corner.

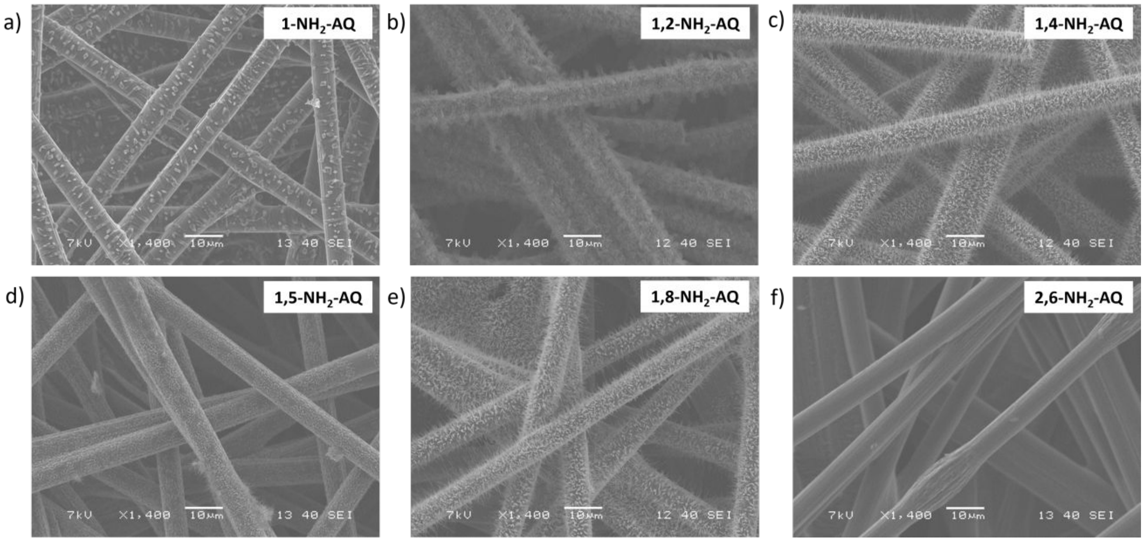

Figure S4. SEM images of the (di-)amino anthraquinone thin-film electrodes.

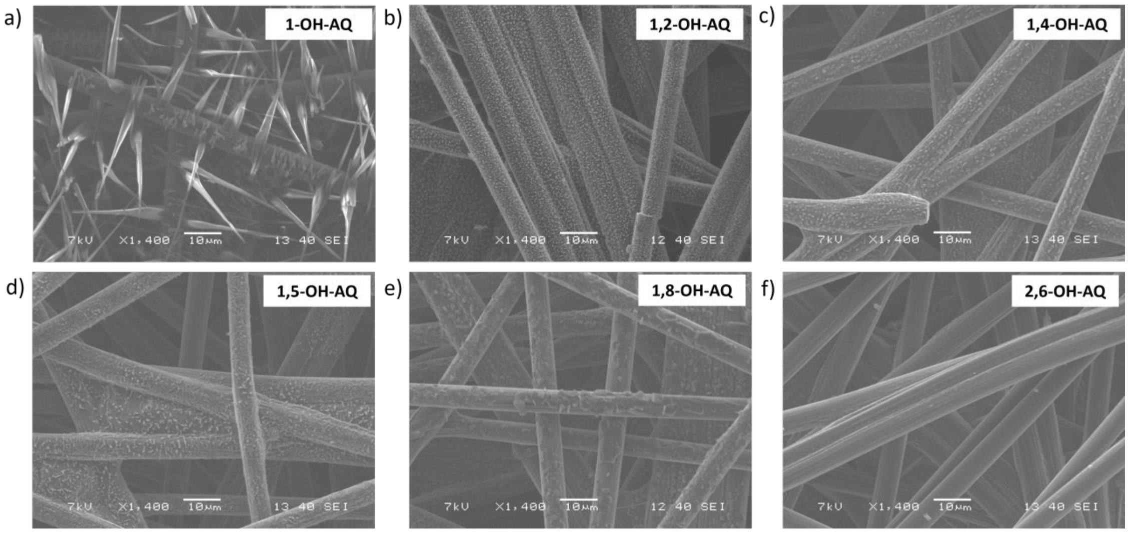

Figure S5. SEM images of the (di-)hydroxy anthraquinone thin-film electrodes.

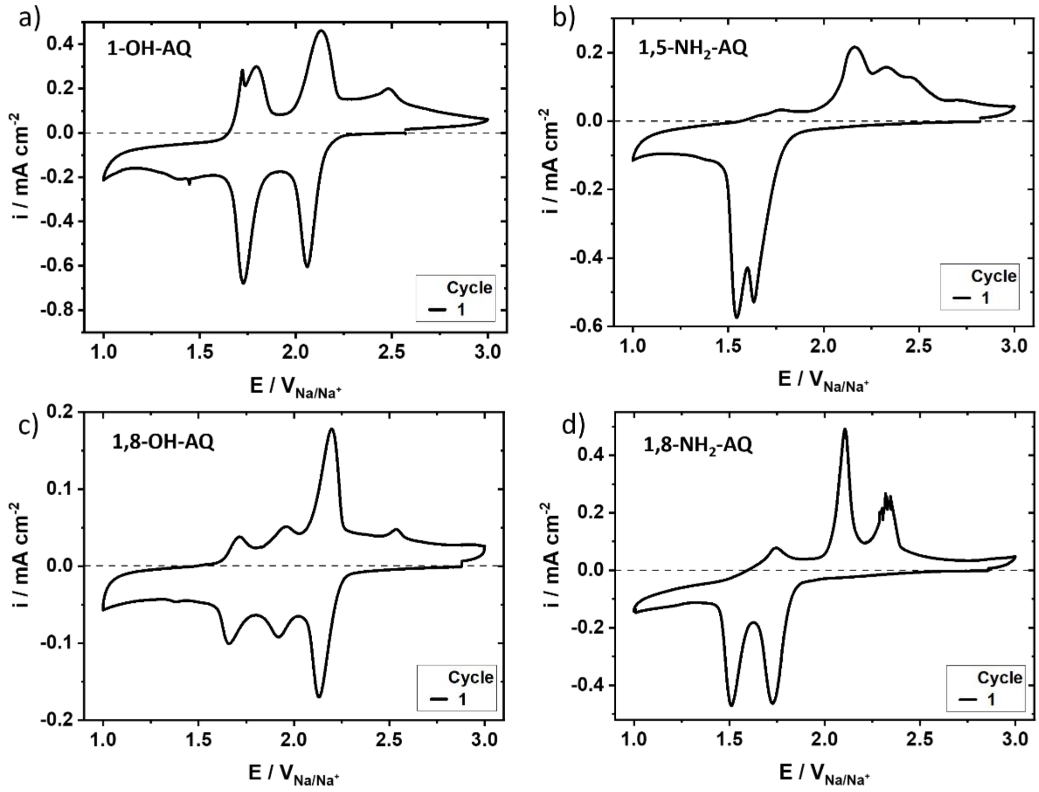

Figure S6. First cycle of the CV measurements of a) 1-OH-AQ, b) 1,5-NH_2-AQ, c) 1,8-OH-AQ and d) 1,8-NH_2-AQ thin-film electrodes with a scan rate of 5mV s^{-1}.

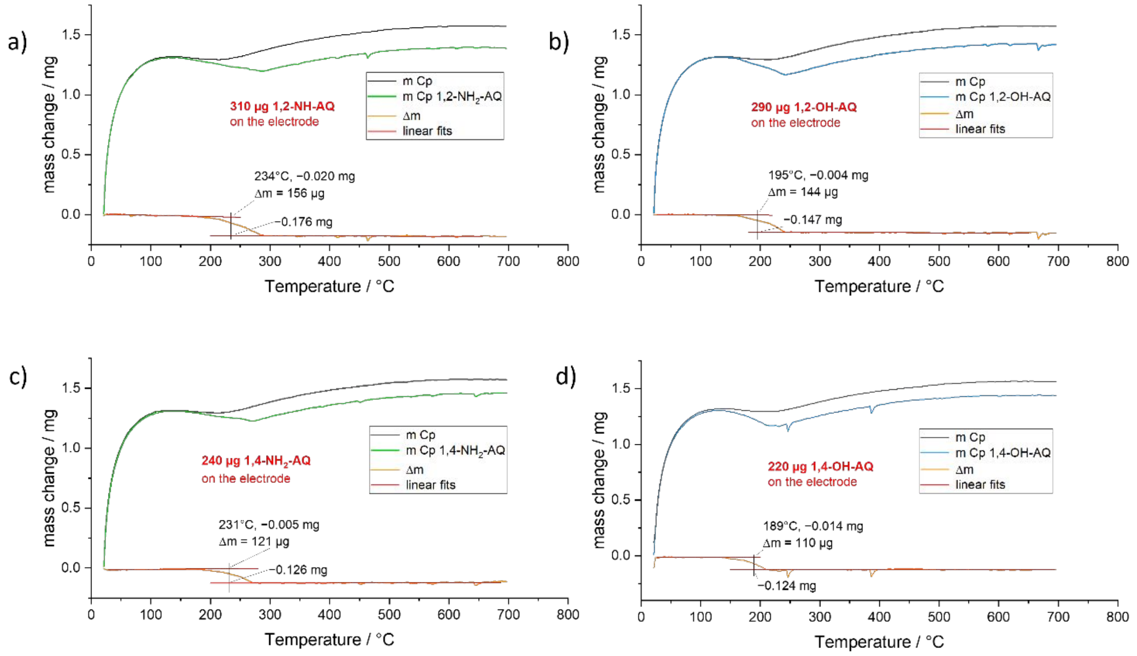

Figure S7: TGA measurements of a) 1,2-NH_2-AQ, b) 1,2-OH-AQ, c) 1,4-NH_2-AQ and d) 1,4-OH-AQ. It is important to note that the measurements of the samples were conducted on only

half of an electrode. Therefore, the mass was multiplied by two in order to calculate the electrode mass.

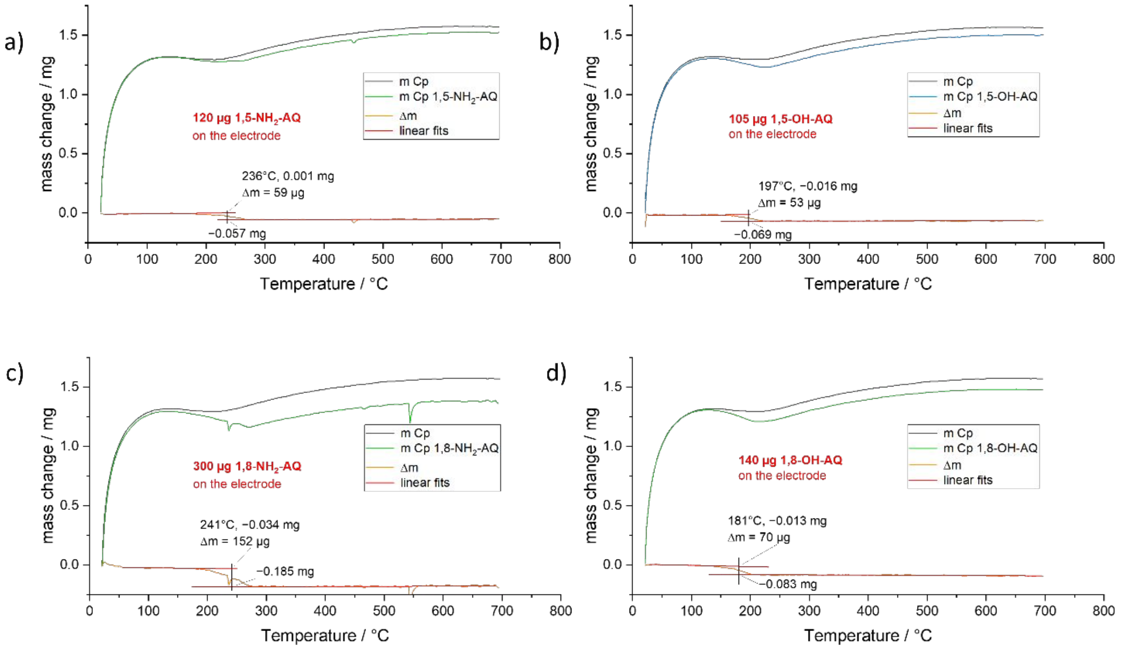

Figure S8. TGA measurements of a) 1,5-NH_2-AQ, b) 1,5-OH-AQ, c) 1,8-NH_2-AQ and d) 1,8-OH-AQ. It is important to note that the measurements of the samples were conducted on only half of an electrode. Therefore, the mass was multiplied by two in order to calculate the electrode mass.

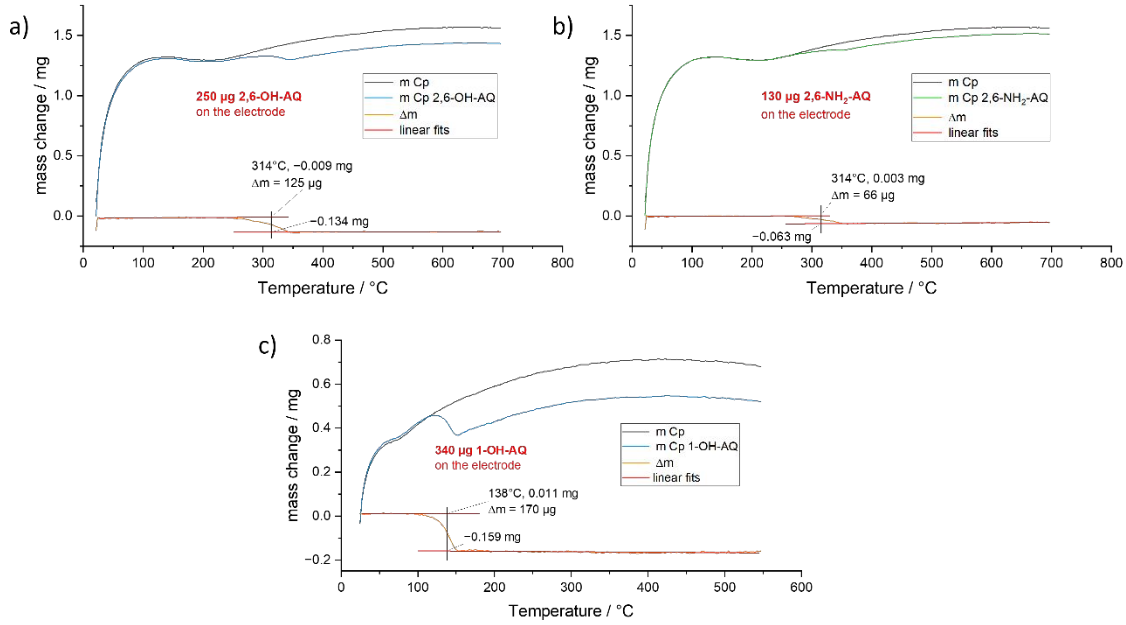

Figure S9. TGA measurements of a) 2,6-OH -AQ, b) 2,6- NH_2-AQ and c) 1-OH-AQ. It is important to note that the measurements of the samples were conducted on only half of an electrode. Therefore, the mass was multiplied by two in order to calculate the electrode mass.

Table S1: The mass loading of the electrodes for the different substituted anthraquinones, calculated with the TGA measurements from S7-S9, the capacities of cycle 1 and cycle 100 and the calculated capacity retention.

Electrode mass / µg	Derivative	capacity cycle 1 / mAh g^{-1}	capacity cycle 100 / mAh g^{-1}	capacity retention / %
310	1,2-NH_2-AQ	47	16	34
290	1,2-OH-AQ	54	23	43
240	1,4-NH_2-AQ	85	30	35
220	1,4-OH-AQ	46	21	46
120	1,5-NH_2-AQ	50	33	66
105	1,5-OH-AQ	148	52	35
300	1,8-NH_2-AQ	52	38	73
140	1,8-OH-AQ	75	55	73
130	2,6-NH_2-AQ	82	39	48
250	2,6-OH-AQ	49	22	45
79	1-NH_2-AQ	54	30	56
340	1-OH-AQ	96	63	66

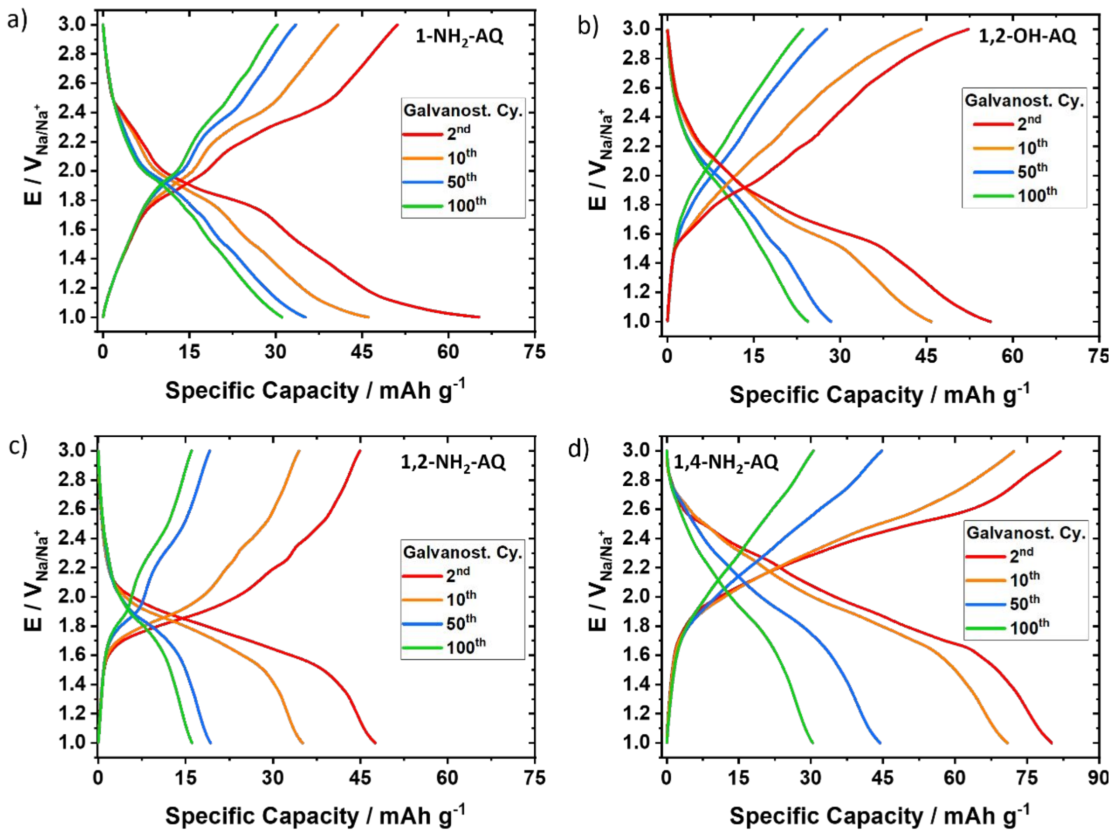

Figure S10. Galvanostatic charge/discharge performances of a) 1-NH_2-AQ, b) 1,2-OH-AQ, c) 1,2-NH_2-AQ and d) 1,4-NH_2-AQ thin-film electrodes for 100 cycles with an applied constant current of 1C.

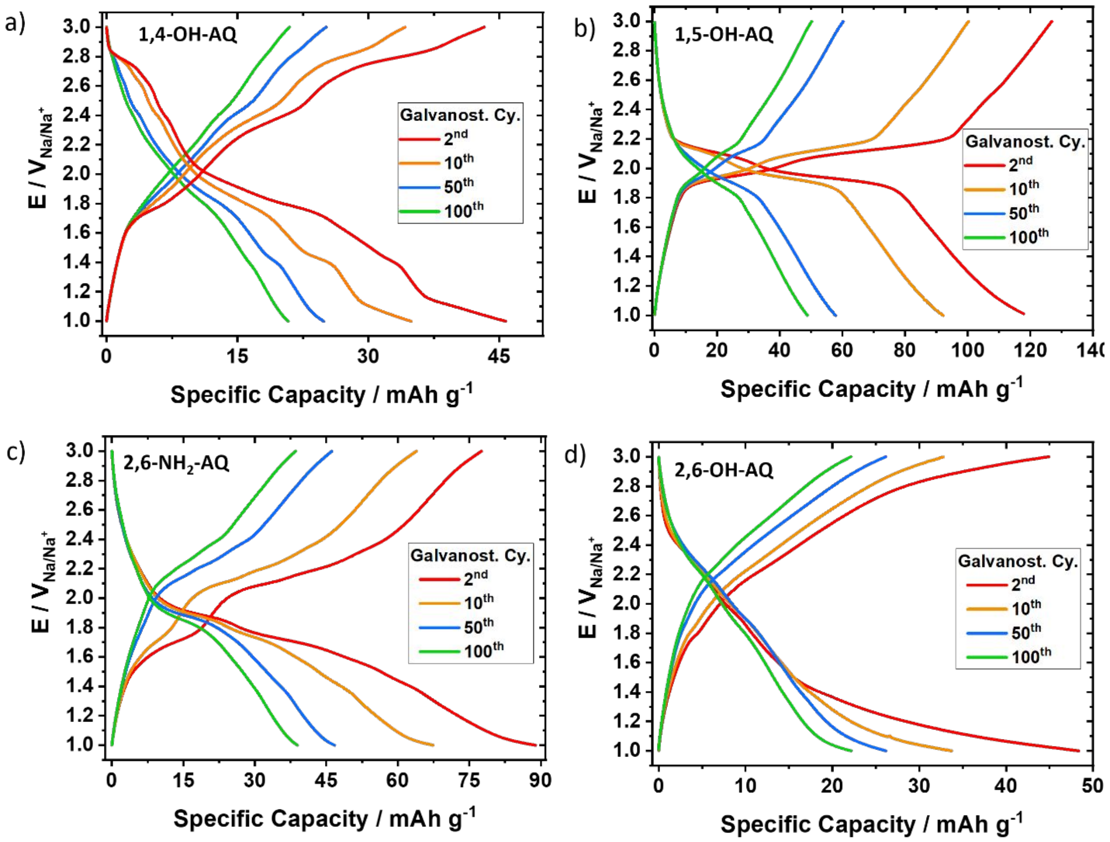

Figure S11. Galvanostatic charge/discharge performances of a) 1,4-OH-AQ, b) 1,5-OH-AQ, c) 2,6-NH$_2$-AQ and d) 2,6-OH-AQ thin-film electrodes for 100 cycles with an applied constant current of 1C.

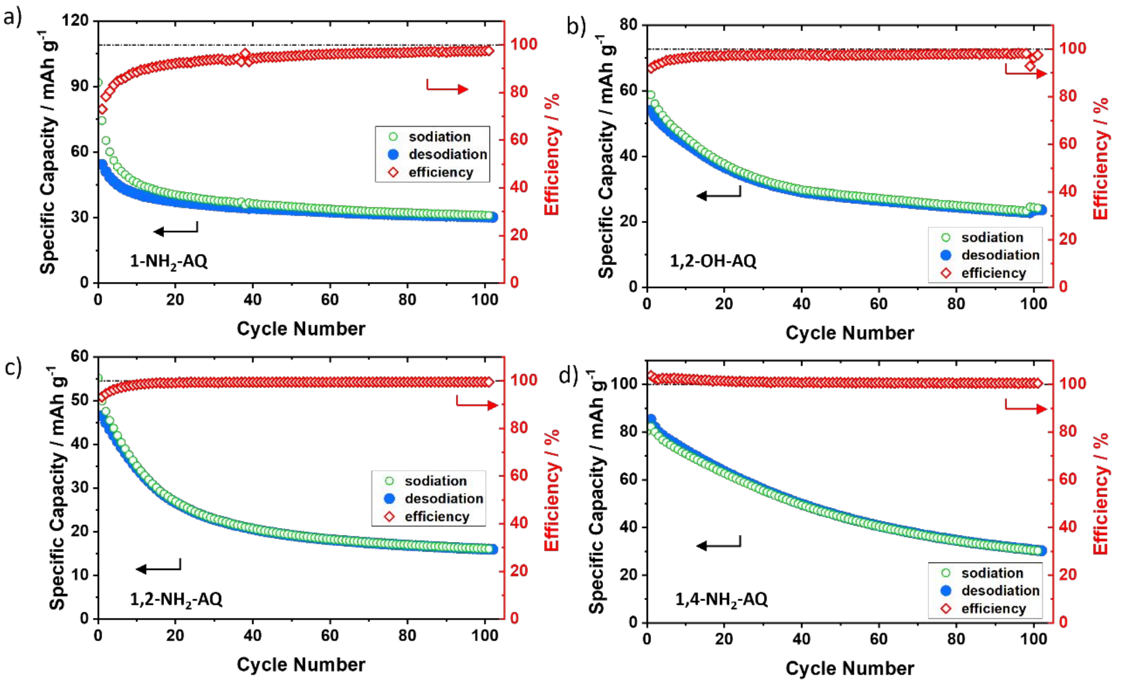

Figure S12. Capacity Retention of a) 1-NH$_2$-AQ, b) 1,2-OH-AQ, c) 1,2-NH$_2$-AQ and d) 1,4-NH$_2$-AQ thin-film electrodes for 100 cycles with an applied constant current of 1C.

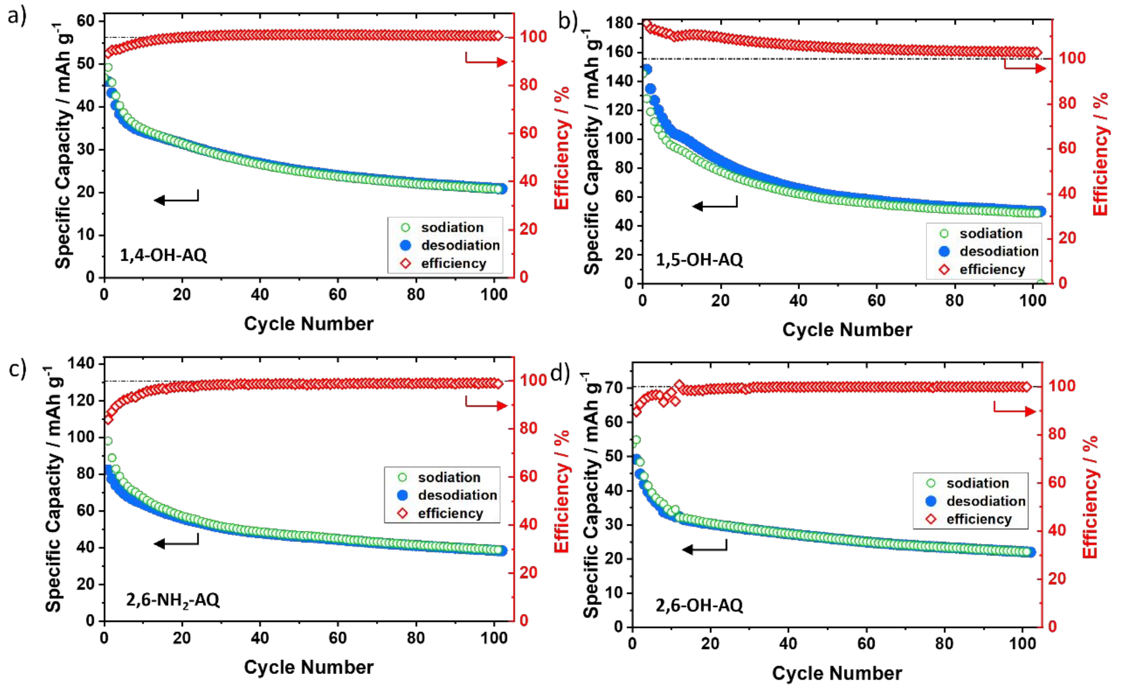

Figure S13. Capacity Retention of a) 1,4-OH-AQ, b) 1,5-OH-AQ, c) 2,6-NH$_2$-AQ and d) 2,6-OH-AQ thin-film electrodes for 100 cycles with an applied constant current of 1C.

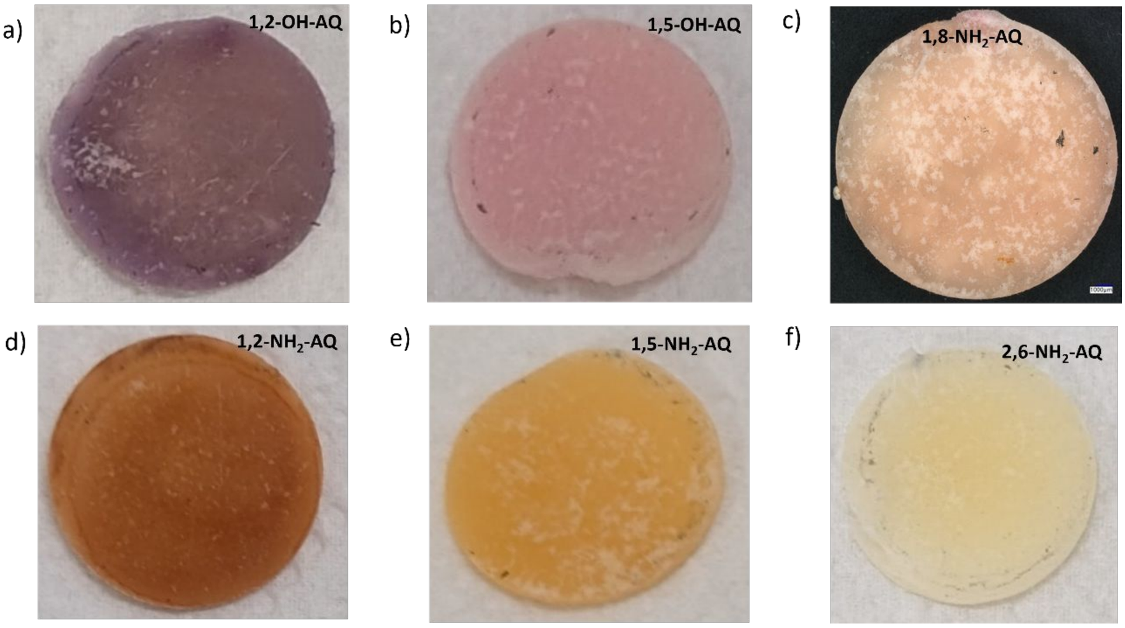

Figure S14. White glass fiber separator disc (Ø = 18 mm, thickness 1.55 mm, El-Cell) after CV and GCPL measurements of six different AQ derivatives.

Bibliography

(1) Dahan, A.; Ashkenazi, T.; Kuznetsov, V.; Makievski, S.; Drug, E.; Fadeev, L.; Bramson, M.; Schokoroy, S.; Rozenshine-Kemelmakher, E.; Gozin, M. Synthesis and Evaluation of a Pseudocyclic Tristhiourea-Based Anion Host. **2007**. https://doi.org/10.1021/jo061774m.
